# Editorial: Exercise and childhood cancer

**DOI:** 10.3389/fped.2022.1097836

**Published:** 2022-11-28

**Authors:** David Mizrahi, Amanda Wurz, Miriam Götte

**Affiliations:** ^1^ The Daffodil Centre, The University of Sydney, a Joint Venture with Cancer Council NSW, Sydney, NSW, Australia; ^2^ Prince of Wales Clinical School, UNSW Medicine & Health, UNSW Sydney, Sydney, NSW, Australia; ^3^ School of Kinesiology, University of the Fraser Valley, Chilliwack, BC, Canada; ^4^ Faculty of Kinesiology, University of Calgary, Calgary, AB, Canada; ^5^ Department of Pediatric Hematology/Oncology, Pediatrics III, West German Cancer Center, University Hospital Essen, Essen, NRW, Germany

**Keywords:** exercise, physical activity, childhood cancer, pediatric oncology, cancer

**Editorial on the Research Topic** Exercise and childhood cancer

Globally, >175,000 children (i.e., individuals aged ≤18–21years) are diagnosed with cancer each year (1). For some of the most common cancers (e.g., acute lymphoblastic leukemia, non-Hodgkin lymphoma), medical advances have led to improved survival rates, with up to 85% expected to survive the disease for at least 5-years (1).^1^ Despite improving prognoses, most children affected by cancer experience, or are at elevated risk, for numerous negative effects during and after treatment, with some effects observed decades later (i.e., late effects) (2). Health behaviors, including physical activity (any movement requiring energy expenditure) and exercise (structured or planned physical activity), may help this cohort manage the effects of their disease during and beyond treatment (3). In fact, based on a burgeoning evidence base, guidelines have recently been published suggesting that all children affected by cancer “move more” (4, 5).

**Figure 1:**
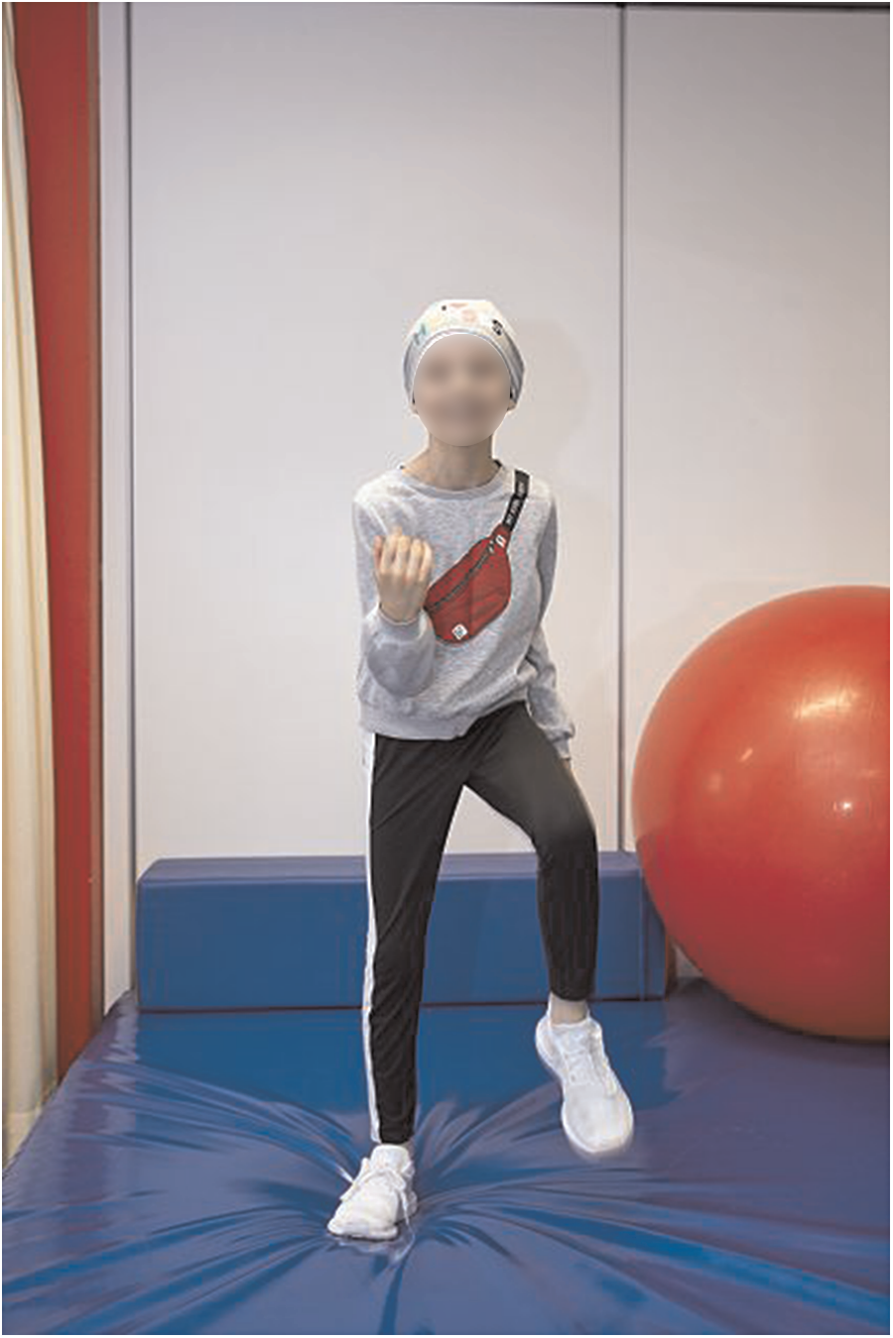
Participant from a pediatric exercise oncology research study.

This Research Topic, *Exercise and Childhood Cancer*, includes 11 articles that collectively advance the field of “pediatric exercise oncology” (6), an area of study exploring physical activity, including exercise, for children affected by cancer. The articles included summarize the current literature, provide further evidence supporting the role of physical activity and exercise for children affected by cancer, and offer insights into forthcoming trials and novel approaches in the field.

## The past: summarizing prior research

Prior published literature reviews have deemed physical activity, including exercise, as safe and potentially beneficial for children affected by cancer, regardless of stage along the cancer trajectory (4–6). However, few efforts have been made to summarize the evidence for children diagnosed with solid and brain tumors, who have historically experienced poorer outcomes (1). Further, few efforts have been made to explore physical activity among those experiencing long-term and late-effects (e.g., cardiotoxicity), which is problematic since this subgroup may need movement more. Thus, we are pleased to share two articles in this Research Topic that address these gaps. Kohler and colleagues conducted a systematic scoping review of 17 studies. The authors concluded that physical activity and exercise interventions were safe, feasible, and seemingly beneficial for children who had completed treatment for brain and other solid tumors. Caru and Curnier provide an in-depth summary of challenges and the potential therapeutic role of integrating physical activity into cancer care to address cancer-related cardiotoxicity in this population.

## The present: advancing the field

The field of pediatric exercise oncology, though still relatively recent, is at an exciting stage with an increasing number of studies published each year. Within this Research Topic, we share two original articles covering innovative interventions, study designs, and approaches to collect data. We also share three articles that explore novel relationships between physical activity (including exercise) and topical outcomes.

In their study with 10 children (6–14 years) who had completed treatment for acute lymphoblastic leukemia, Marchese and colleagues found a 6-week targeted exercise program (supervised and home-based progressive beaded jumping rope) improved gross motor performance and augmented observed neuromuscular impairments, which could offer a low-cost intervention for at-home and in-school. The authors assessed neuromuscular outcomes with tools that have rarely, if ever, been utilized in this population (e.g., electromyography, motion capture, force plates). In their randomized controlled trial (RCT), Gaser and colleagues assigned 41 children and adolescents (4–18 years), who were receiving treatment for leukemia or non-Hodgkin lymphoma, to an individualized resistance intervention or standard exercise (sports games, aerobic or coordination exercises) for an average of 7.4 months. This is among the first superiority RCT in the field, with findings suggesting both groups experienced improvements in activities of daily living, though the resistance intervention was more beneficial for explosive strength.

The observational studies included herein provide early evidence that physical activity, including exercise, may provide important short and long-term health benefits. Specifically, Bratteteig and colleagues examined the association between device-measured physical activity intensities and cardiovascular disease risk among 157 children (9–18 years) who had completed treatment ≥1 year prior. Vigorous physical activity was associated with clinically meaningful improved cardiovascular disease risk profiles, suggesting monitoring and promoting vigorous physical activity may be important for long-term cardiovascular health. Among 185 adults previously diagnosed with cancer as a child, Goodenough and colleagues found that p16^INKa^ (a biomarker of cellular senescence) and inflammation, were associated with lower exercise capacity. These findings offer possible targets to remediate some of the adverse physiologic outcomes observed in this population. In their study of 1,166 adults who had been diagnosed with acute lymphoblastic leukemia as a child, Wogksch and colleagues found that being obese was associated with a higher energy cost when walking, worse adaptive physical function, and lower health-related quality of life than controls. These findings suggest weight loss interventions may be important as a means of supporting the growing population of adults who were diagnosed with cancer as a child.

## The future: work in preparation and progress

In this Research Topic, we are able to look ahead to planned and ongoing work, which will undoubtedly advance the field of pediatric exercise oncology. Indeed, the included articles address recently identified research and innovation needs (4). Specifically, Schmidt-Andersen and colleagues are conducting the INTERACT trial, a multi-site study that will recruit 127 newly diagnosed children (6–17 years) and randomize them into 6-months of supervised (and home-based) integrative neuromuscular exercise or an unsupervised active usual care group (resistance, aerobic and stretching exercises). The primary outcome explored will be muscular strength, with findings aiming to support adapted interventions for hospital and home settings. The same research group led by Pouplier and colleagues will randomize 84 newly diagnosed pre-school aged children (1–5 years) to a combined intervention with in-hospital, structured active play and home-based active play, or usual care, and explore effects on gross motor function and social and personal skills. Findings will contribute vital evidence, not only for this subgroup within the childhood cancer population who are understudied, but for the efficacy of play-based interventions. Of note, this trial will utilize the RePlay Model, a conceptual model for structured active play for pre-schoolers created by the same study team and published herein (i.e., Pouplier et al.). Finally, in their paper Grimshaw and colleagues developed a behavior change intervention entitled: CanMOVE for school-aged children undergoing treatment (5–16 years), which incorporates 15 strategies designed to promote and increase physical activity.

Collectively, the studies published in this Research Topic address gaps in knowledge and highlight innovations in research seeking to promote physical activity, including exercise, among children affected by cancer. While much remains to be discovered, these findings bring us one step closer to ensuring all children affected by cancer have the opportunity to improve their health by moving more (Figure 1).

## Footnotes

^1^Prognosis varies across cancer types and is lower in middle- and low-income countries.
